# The factor structure of major depressive symptoms in a sample of Chinese earthquake survivors

**DOI:** 10.1186/s12888-020-02993-3

**Published:** 2021-01-28

**Authors:** Yajie Bi, Li Wang, Chengqi Cao, Ruojiao Fang, Gen Li, Ping Liu, Shu Luo, Haibo Yang, Brian J. Hall

**Affiliations:** 1 Laboratory for Traumatic Stress Studies, CAS Key Laboratory of Mental Health, Institute of Psychology, Chinese Academy of Sciences, 16 Lincui Road, Beijing, 100101 China; 2 Department of Psychology, University of Chinese Academy of Sciences, Beijing, China; 3 Shenzhen Key Laboratory of Affective and Social Cognitive Science, Shenzhen University, Shenzhen, China; 4 People’s Hospital of Deyang City, Deyang, Sichuan China; 5 Academy of Psychology and Behavior, Tianjin Normal University, Tianjin, China; 6 Global and Community Mental Health Research Group, New York University (Shanghai), Pudong, Shanghai, China

**Keywords:** Major depressive disorders, Confirmatory factor analysis, Health-related quality of life, Chinese, Disaster

## Abstract

**Background:**

Experiencing natural disasters is associated with common mental disorders including major depressive disorder (MDD). However, the latent structure of MDD is widely debated, and few studies tested the MDD factor structure in Chinese natural disaster survivors. Therefore, the aim of the current study was to evaluate the factorial validity of the Patient Health Questionnaire-9 (PHQ-9) for DSM-5 major depressive disorder (MDD) symptoms in Chinese earthquake survivors.

**Method:**

Participants were 1058 Chinese earthquake survivors. Self-reported measures included the PHQ-9 and the Short-Form Health Survey (SF-36). Confirmatory factor analysis (CFA) and structural equation modelling (SEM) was used to examine the latent structure of MDD and the associations between latent factors of MDD and different domains of health-related quality of life (HRQoL), respectively.

**Results:**

In the current sample, the model consisted of somatic and cognitive/affective (non-somatic) factors demonstrated significantly better fit than the other competing MDD models (*χ*^2^ = 173.89, *df* = 26, CFI = 0.986, TLI = 0.981, RMSEA = 0.073, BIC = 18,091.13). Further SEM analyses indicated that the non-somatic factor was significantly related to both physical (*β* = − 0.362, *p* < .01) and psychosocial HRQoL (*β* = − 0.773, *p* < .01), while the somatic factor was a uniquely predictor of physical HRQoL (*β* = − 0.336, *p* < .01). Furthermore, we found the somatic factor partially mediated the relationship between the cognitive/affective factor and physical HRQoL (all *p*s < .05).

**Conclusions:**

The MDD symptoms was best captured by a two-factor model comprised of somatic and cognitive/affective factors in Chinese natural disaster survivors. The two MDD factors were differentially associated with physical and psychosocial HRQoL, and the cognitive/affective factor associated physical HRQoL partially through the somatic factor. The current findings increase our understanding of latent structure of MDD symptoms, and carry implications for assessment and intervention of post-disaster mental health problems.

## Background

Due to urbanization, environmental degradation, climate change and other factors, the frequency of natural disasters has increased dramatically in recent years [1]. Natural disasters not only cause economic loss, injuries and deaths [2], but also cause severe psychological distress and psychiatric disorders [3]. Major depressive disorder (MDD) is one of the most common psychiatric disorders in natural disaster-exposed populations [4], and MDD symptoms increase with time among natural disaster-exposed individuals [5]. Compared to the prevalence of MDD in general population of 4.7% [6], natural disaster-exposed populations range from 10.0–17.6% [7–10]. Therefore, it is important to develop accurate assessment that guides effective prevention and intervention for MDD after natural disaster. Exploring the factor structure of MDD symptoms could not only benefit our understanding of psychopathological and biological mechanisms, but is also important for the development of more sophisticated clinical assessment and effective treatment programs for this disorder. The PHQ-9 is a self-report questionnaire and maps onto the criteria of MDD in *DSM-5* [11]. The reliability and validity of PHQ-9 have been evaluated in numerous populations globally [12, 13]. The PHQ-9 is one of the most effective and commonly used instrument for measuring depression [14], however, the structure of MDD measured by PHQ-9 is debated.

Several alternative models of MDD symptoms has been proposed and validated in previous studies [13, 15–17] (see Table 1). Model 1 is the unidimensional model based on PCA/CFA studies of primary care patients [18, 19] and spinal cord injury patients [20]. A number of studies in various samples (e.g., Chinese adults and adolescents, Mexican women, American college students) have found robust evidence for the unidimensional model that underlies the PHQ-9 [13, 21–23], and many of them excluded two-factor models for the high correlation between MDD factors [22–24]. Other competitive models are similar and comprised of 2 factors: a somatic factor and a cognitive/affective (non-somatic) factor. Krause et al. (2008, 2010) measured MDD of spinal cord injury patients at an average of 50 days after injury, and proposed the model 2a in CFA studies [15, 25]. This finding was confirmed by a longitudinal study of palliative care patients [26]. Model 2b was based on a CFA study of coronary heart disease patients [16]. Model 2c was proposed in EFA and CFA studies among spinal cord injury patients [25, 27], and was confirmed by the CFA studies in soldiers [17], primary care patients in Germany [28], Filipino and Indonesian female domestic workers in China [29, 30], mental health patients [31] and university students in Colombia [22]. Model 2d was first described in the sample of spinal cord injury patients at 30 months after injury [25]. Notably, Model 2c has been confirmed in more studies than the other competing models [17, 28–30], and this may be attributed to type of populations, demographics, health condition, and history of trauma exposure [29].

**Table 1:** Item mapping for confirmatory factor analysis

PHQ-9 Items	Model 1	Model 2a	Model 2b	Model 2c	Model 2d
Anhedonia	Depression	Non-somatic	Non-somatic	Non-somatic	Somatic
Depressed mood	Depression	Non-somatic	Non-somatic	Non-somatic	Non-somatic
Sleep difficulties	Depression	Somatic	Somatic	Somatic	Somatic
Fatigue	Depression	Somatic	Somatic	Somatic	Somatic
Appetite changed	Depression	Somatic	Somatic	Somatic	Somatic
Feeling of worthless	Depression	Non-somatic	Non-somatic	Non-somatic	Non-somatic
Concentration difficulties	Depression	Non-somatic	Non-somatic	Somatic	Somatic
Psychomotor agitations/retardation	Depression	Non-somatic	Somatic	Somatic	Somatic
Thoughts of death	Depression	Non-somatic	Non-somatic	Non-somatic	Non-somatic

Model 1 posits that a single one-dimension factor underlies the PHQ-9; Model 2a: Krause et al. [15], Krause et al. [25], Chilcot et al. [26]; Model 2b: de jonge et al. [16]; Model 2c: Krause et al. [25], Richardson & Richards, [27], Elhai et al. [17], Petersen et al. [28], Mordeno et al. [29], Guo et al. [31], Miranda & Scoppetta [32]. Model 2d: Krause et al. [25]

Many studies have explored the factor structure of MDD, while few of them clarified the different functional roles of MDD symptom clusters. Simms (2002) suggested that external measures of psychopathology should be examined to clarify this issue [33]. As highlighted by several studies, MDD is tightly associated to poor HRQoL [34], and QoL is an important consideration of treatment-outcome assessment in MDD patients [35]. Previous studies suggested that somatic symptoms (i.e., sleep disturbance, fatigue and appetite change) were uniquely associated with reduced physical health [36, 37]. Furthermore, some non-somatic symptoms such as feeling worthless and suicidal ideas might be related closely to poor psychosocial functioning [38, 39], and could reduce physical health [40]. Therefore, the discriminant validity of the best fitting model can be examined by estimating the correlations between factors of MDD and external measures of HRQoL.

Previous studies revealed that people who suffer from MDD often had physical problems [41, 42], and some psychotherapies (e.g., cognitive therapy) have positive effects on the physical problems of MDD patients [43]. Therefore, investigations are needed that document the association between MDD symptoms and physical HRQoL. However, few studies discussed this issue. Non-somatic and somatic depression factors might be related to physical HRQoL. Within traditional Chinese culture, people are reluctant to express their emotions and psychological distress. They tend to attribute psychological problems to physical or external origins [44]. Therefore, cognitive/affective symptoms of MDD might be expressed through somatic symptoms in Chinese samples, which may in turn affect physical HRQoL.

The first aim of this study was to find the best fitting latent structural model of MDD; our second aim was to evaluate whether the somatic factor mediates the relationship between non-somatic factor and physical HRQoL. According to prior studies, our hypotheses were as follows: (1) the model comprised of a somatic factor and a cognitive/affective factor would provide a superior data fit over competing models; (2) the non-somatic factor would be associated with psychosocial health and physical health. Relatedly, we expected the somatic factor would correlate with physical health only; and, (3) the non-somatic factor would affect physical HRQoL through the somatic factor. Many previous study samples were restricted to medical patients (e.g. patients with spinal cord injury, coronary heart disease patients), which might increase the endorsement rates of the MDD somatic symptoms and therefore might affect the factor structure of MDD [15, 16, 25, 27]. Our study is the first one to use disaster-exposed sample, which could examine the generalizability of previous results and extend current knowledge on MDD symptom structure.

## Methods

### Procedure and participants

On May 12, 2008, a destructive earthquake measuring 8.0 on the Richter scale occurred in Sichuan Province. For the purposes of accessing disaster-related mental health problems, the sample was collected from 5 rebuilt communities of Hanwang town, Mianzhu City. Approximately 5000 people living in this town were killed in the earthquake. This survey was conducted in July, 2017. The procedures were as follows: (1) Selecting one family member randomly in each household as participants; (2) making sure that all participants were aged from 16 to 65 and experienced the 2008 earthquake; (3) excluding individuals with mental retardation or a major psychiatry history. This survey was conducted by professional clinical psychologists, psychology students. Before the participants completed questionnaires, the aim of the study was clearly explained. All participants have signed an informed consent. This study protocol was reviewed and approved by the Institutional Review Board of the Institute of Psychology, Chinese Academy of Sciences. A total of 1072 people participated in this study. People with missing data (> 20% items) were excluded (*n* = 14), leaving an effective sample of 1058.

### Measures

MDD symptoms were accessed using the PHQ-9, which is a nine-item instrument from the Primary Care Evaluation of Mental Disorders (PRIME-MD). PHQ-9 is a brief, easily administered self-report questionnaire designed to capture MDD symptoms. Each item is rated on a 4-point scale using anchors ranging from 0 (not at all) to 3 (extremely), reflecting the extent which symptom bothers a participant in the past 2 weeks. The reliability and validity of the PHQ-9 have been well-documented in a previous study [14]. The PHQ-9 has been translated to more than 80 languages and widely used worldwide, and the Chinese version of PHQ-9 has been reported to have good psychometric properties [12]. Cronbach’s α for PHQ-9 was 0.89 in the current sample.

The Short-Form Health Survey (SF-36) was used to assess health-related quality of life. There are 8 subscales in this instrument, 4 reflect Physical HRQoL (consisting of physical functioning, role-physical, bodily pain and general health), and 4 reflect Psychological HRQoL (consisting of role-emotional, social functioning, mental health and fatigue). Scores of all subscales range from 0 to 100, with higher scores indicating a better HRQoL. The Chinese version of SF-36 has been proven to have good reliability and validity, and widely used in Chinese populations [45]. Wang (2014) identified that the PHQ-9 total score correlated negatively with each subscale’s score of the SF-36 [12]. We select scales of SF-36 because their content can explicitly address psychosocial functioning. Mental health and Fatigue subscales were excluded for the observation of spurious relationship with the MDD measures.

### Statistical analysis

All descriptive analyses were conducted using the SPSS 20.0 for Windows. Of the 1058 participants, 36 were missing one PHQ-9 item, 284 were missing one to three SF-36 items and 6 were missing four to six SF-36 items. Missing values on the PHQ-9 were handled with full information maximum likelihood (ML) procedures. According to the scoring procedures of the SF-36, the missing values on the SF-36 were estimated with the average score of the same subscales.

To evaluate the best-fitting factor model, five alternative CFA models (see Table 1) previously identified were tested in the current study. According to previous studies, items should be treated as categorical variables when measures contain fewer than five options, and Weighted Least Squares Estimation with a mean and variance adjusted (WLSMV) should also be used [46]. Therefore, all measurement models were evaluated using CFA and WLSMV in Mplus 7.0. Three conventional indices (CFI, TLI and RMSEA) were used to evaluate the internal fit of the models. For these indices, an excellent fit is indicated by CFI and TLI of 0.95 and above, RMSEA of 0.06 or less, and an acceptable fit is indicated by CFI and TLI of 0.90 and above, RMSEA of 0.08 or less [47]. Chi-square difference tests were conducted to compare nested models using the DIFFTEST function in Mplus. The Bayesian information criterion (BIC) were used to compare non-nested models. A BIC difference of 6–10 provides strong support and a difference greater than 10 indicates very strong support for the model with the lower BIC value [48]. However, the BIC can only be generated by ML, therefore, we use maximum likelihood estimation with robust standard (MLR) to compute this index.

We subsequently incorporated two structural equation models to evaluate the correlations between each MDD factor and each component of HRQoL. In the first model, physical health was treated as a latent factor measured by the physical functioning, role-physical, bodily pain, and general health subscales of SF-36 subscales (for estimation of latent variables could reduce the measurement error compared with summing sub-scales’ scores), and was set as the dependent latent variable. In the second model, psychosocial quality of life was treated as a latent factor measured by the role-emotional and social functioning, and was set as the dependent latent variable. MDD factors of the best fitting model and covariates (age, gender, educational level and marital status) were simultaneously included in the equation as predictors in all models. SEM was used to test all models, and regression coefficients were calculated to examine the different correlations between each MDD factor with physical and psychosocial HRQoL.

A SEM model was built to examine the mediator role of MDD’s somatic factor between MDD’s non-somatic factor and physical HRQoL, standard errors of the mediating parameter were simultaneously estimated in the SEM model with bootstrap procedure (1000 times). Direct effects in this study include the relationship between MDD’s somatic and cognitive/affective factors, between MDD’s somatic factors and physical health, and between MDD’s non-somatic factors and physical health. Indirect effects included the effect of MDD’s cognitive/affective factor on physical health through MDD’s somatic factor.

## Results

### Descriptive statistics

In the current sample, 347 (32.7%) men and 711 (67.3%) women with a mean age of 51.1 (range: 16–65, *SD* = 10.0), 911 (86.2%) were married, and 146 (13.8%) were unmarried (consisting of never married, divorced, widowed). 1042 (98.6%) were Han people, and 5 (0.4%) were members of other ethnic groups (including Zang, Qiang and Hui). 732 (69.2%) did not complete high school, 240 (22.7%) completed high school, and 85 (8.0%) completed college.

Descriptive statistics for the PHQ-9 and SF-36 total scores were estimated at first. Mean PHQ-9 score was 6.2 (range: 0–27, *SD* = 5.0), and 236 (22.2%) participants were diagnosed with possible MDD (based on the diagnostic algorithm of using a cutoff score of 10 or higher).

Mean scores on the SF-36 subscales were as follows: “Physical Functioning” was 79.2 (*SD* = 24.2, range: 0–100), “Role limitations due to physical health” was 64.4 (*SD* = 41.0, range: 0–100), “Bodily Pain” was 76.4 (*SD* = 22.3, range: 0–100), “General Health” was 60.7 (*SD* = 21.2, range: 0–100), “Social Functioning” was 79.6 (*SD* = 21.4, range: 0–100), and the “Role limitations due to emotional problems” was 60.3 (*SD* = 41.6, range: 0–100).

### Comparison of competitive models

**Fig. 1:**
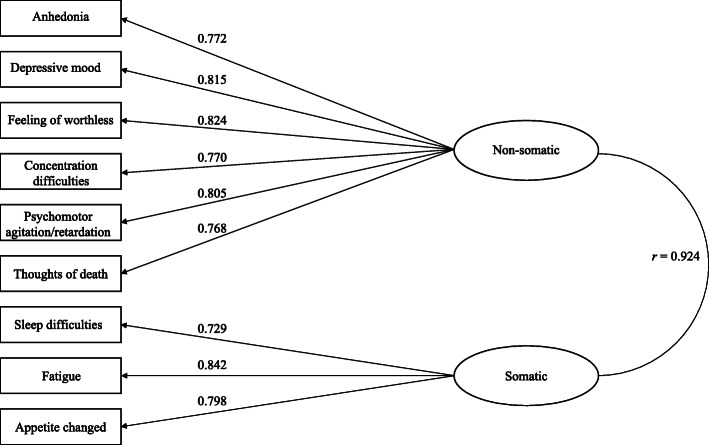
The two-factor model of MDD symptoms assessed by PHQ-9. *Note. N* = 1058; All correlations are statistically significant (*p* < 0.01)

Table 2 presents internal fit indices of alternative models. Only Model 2a yielded acceptable fit, while other models were rejected due to large RMSEA values. Table 3 displays results of Chi-square difference tests for nested models, only Model 2a was superior to model 1 (△*χ*^2^ (1) = 45.653, *p* < 0.001). In terms of comparisons of non-nested models, the BIC value for model 2a was lower than the other models, and all △BIC were lower than 6 except the △BIC between Model 2a and other models. For these reasons, the Model 2a was the best fitting model (see Tables 2 and 3). The standardized factor loadings and factor intercorrelations of Model 2a were summarized in Fig. 1.

**Table 2:** Model goodness of fit indices

Model	*χ*^2^	*df*	CFI	TLI	RMSEA	RMSEA 90% CI	BIC
Model 1	239.38	27	0.980	0.974	0.086	0.076–0.096	18,129.67
Model 2a	173.89	26	0.986	0.981	0.073	0.063–0.084	18,091.13
Model 2b	233.96	26	0.981	0.974	0.087	0.077–0.097	18,134.56
Model 2c	235.07	26	0.981	0.973	0.087	0.077–0.098	18,135.16
Model 2d	237.65	26	0.981	0.973	0.088	0.077–0.098	18,133.81

*N* = 1058. *CFI* comparative fit index, *TLI* Tucker-Lewis index, *RMSEA* root mean square error of approximation, *CI* confidence interval, *BIC* Bayesian information criterion

**Table 3:** Chi-square difference test for comparing nest models

Models	△*χ*^2^ (*df*)	*p*
Model 1 vs Model 2a	45.65(1)	< 0.001
Model 1 vs Model 2b	6.52(1)	0.011
Model 1 vs Model 2c	5.36(1)	0.021
Model 1 vs Model 2d	2.91(1)	0.088

*N* = 1058

### Correlations between PHQ-9 factors and HRQoL components

The models examining correlations between MDD factors and HRQoL demonstrated adequate fit. Fit statistics were *χ*^2^ (110, *N* = 1058) = 218.08, CFI = 0.99, TLI = 0.99, RMSEA = 0.03 (90% CI: 0.03–0.04) for the model with physical HRQoL as dependent variable, and *χ*^2^ (81, *N* = 1058) = 243.98, CFI = 0.99, TLI = 0.98, RMSEA = 0.04 (90% CI: 0.04 ~ 0.05) for the model with psychosocial HRQoL as a dependent variable. Table 4 summarizes the associations between the two MDD factors and physical and psychosocial HRQoL. After controlling other variables, the somatic factor significantly predicted the physical HRQoL (*β* = − 0.336, *p* < .01), and the cognitive/affective factor was a significant predictor for both physical HRQoL (*β* = − 0.362, *p* < .01) and psychosocial HRQoL (*β* = − 0.773, *p* < .01).

**Table 4:** Relationship between MDD factors and health related quality of life

Dependent variables	Predictor	*r*	*B*	*β*
Physical health-related quality of life	Somatic	−0.674	−5.637*	−0.336*
	Non-somatic	−0.678	−5.687*	−0.362*
Psychosocial health-related quality of life	Somatic	−0.691	0.795	0.036
	Non-somatic	−0.741	−14.955*	−0.733*

*N* = 1058

*p < 0.01

### The mediating effect of the somatic factor

**Fig. 2:**
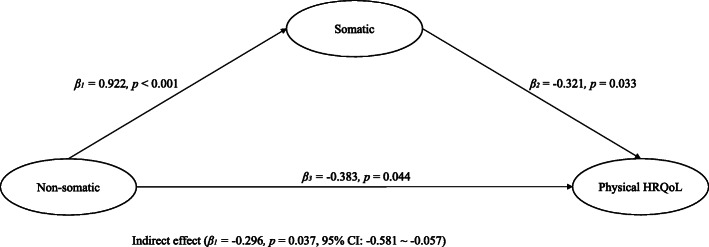
Mediating role of MDD’s somatic factor in the relationship between MDD’s non-somatic and physical health-related quality of life. *Note.* *N *= 1058. HRQol, health-related quality of life

The indirect effect of the non-somatic factor on physical HRQoL was significant (*β* = − 0.296, *p* < .05) (see Fig. 2). Results indicated significant effects of the cognitive/affective factor on the somatic factor (*β*_1_ = 0.922, *SE* = 0.016, *p* < .05) and the somatic factor on physical HRQoL (*β*_2_ = − 0.321, *SE* = 0.153, *p* < .05). The path from cognitive/affective factor to physical HRQoL was also significant (*β*_3_ = − 0.383, *SE* = 0.149, *p* < .05). After 1000 bootstrapped tests, the somatic factor still significantly mediated the relationship (95% CI of *β*_1_: 0.894 ~ 0.957; 95% CI of *β*_2_: − 0.645 ~ − 0.061; 95% CI of *β*_3_: − 0.629 ~ − 0.082; 95% CI of indirect effect: − 0.581 ~ − 0.057).

## Discussion

The current study investigated the factor structure of MDD symptoms measured by PHQ-9 in a sample of Chinese earthquake survivors, found the two-factor model of MDD symptoms is the best fitting model, and MDD factors had different associations with physical and psychosocial HRQoL. Since Krause (2008) proposed that MDD symptoms could be best captured by somatic and cognitive/affective factors, many studies supported two-factor MDD models in samples with somatic conditions (e.g. patients with spinal cord injury, coronary heart disease patients) [15, 16, 25, 27]. However, using samples of patients with somatic conditions might increase endorsement rates of MDD somatic symptoms, and thus might influence the factor structure of MDD. Our findings were consistent with results of previous studies used medical patients [15, 16, 25, 27, 28] and used other samples [17, 29, 30], and support separating the MDD into a somatic factor and cognitive/affective factor. In the current sample, the correlation between two MDD factors was 0.92, this might be criticized for lacking of discriminant validity. However, high correlation between factors cannot be enough to prove that the model lacks discriminant validity. First, the current study and many previous studies have found that the two-factor MDD model had significantly better fitting statistics than the one-factor MDD model [17, 28–30, 49]. In addition, the results yielded from bootstrap analysis showed that the 95%CI of correlation coefficient between somatic and non-somatic factors did not include 1 (0.890–0.953), further justified separating these two factors. These findings indicated that MDD symptoms could be captured by somatic and cognitive/affective factors. Second, Mordeno et al. (2018) have found that somatic symptoms (measured by PHQ-15) were more closely related to the somatic factor than the cognitive/affective factor of MDD [29], and the present study also found the physical and psychosocial HRQoL had different associations with the somatic factor and the cognitive/affective factor of MDD. These findings provided evidence for the external validity of the distinction between the MDD somatic and non-somatic factors. Third, the two-factor model could be better interpreted as different symptomatic representation and subtypes of MDD than the unidimensional model [50, 51]. A number of empirical studies used machine learning, genetic, and biological approaches to yield strong evidence in favor of MDD consisting of somatic and affective/cognitive factors, rather than one “depression” factor [51–53]. It is interesting that the best fitting model in this study contradicted the results of Elhai et al. (2012), Petersen et al. (2015) and Mordeno et al. (2018). The concentrating difficulties and psychomotor agitation/retardation loaded on the cognitive/affective factor in the current study. Papakostas (2013) reported that concentration difficulties and the psychomotor agitation/retardation symptoms reflected deficits in cognitive functions, and subsequent research using the Research Domain Criteria (RDoC) framework confirmed that these two symptoms should be classified in cognitive and sensorimotor systems [53, 54]. Moreover, two recent CFA studies have found similar conclusions in adults with autism and in the general US population [55, 56].

To further verify the distinctiveness of the two MDD’s factors, the current study used HRQoL as an external measure, and found that HRQoL displayed specific associations with two factors of MDD. Previous studies also had similar findings. The results of Mordeno et al., (2018)‘s study revealed that somatic symptoms (measured by PHQ-15) were more related to the somatic factor than cognitive/affective factor [29]. Elhai et al. (2011) also found that in Canadian military veterans measured by Center for Epidemiologic Studies-Depression Scale (CES-D), depression’s somatic items had higher relationship with some factors of posttraumatic stress disorder (PTSD) than non-somatic items [57]. Finally, another study demonstrated that the Australian and UK samples measured by the Gotland Scale of Male Depression (GSMD) were best represented by a two-factor (affective and somatic) MDD model [58]. The results supported our hypotheses, and illustrated that the distinct factors of MDD might play different functional roles in the HRQoL of people with depression and provided further robust support for the two-factor MDD model.

Furthermore, based on previous theoretical evidence, we assessed the mediating role of somatic factor referencing the relationship between the non-somatic factor and physical HRQoL. Results indicated that the non-somatic factor of MDD probably affects the physical HRQoL via the MDD somatic factor in the present sample. This may have a cultural explanation. For example, expressing psychological distress is considered a dilemma in Chinese culture. People who experience psychological problems tend to attribute these problems to physical or external origins [44, 59]. This might be the reason that psychological problems were often expressed in the form of physical illness in Asian populations. Our findings partially clarified the pattern of MDD’s non-somatic factor influencing the physical HRQoL in the Chinese natural disaster survivors.

Finding of this study has several implications for clinical applications. First, investigation of the factor structure of MDD could help us better understanding different components of MDD symptoms, which could guide clinically useful assessment and intervention for MDD symptoms. Second, findings of our study informed the different functional roles that distinct factors of MDD played in HRQoL. This would be helpful to structure and establish treatment which aims to improve the HRQoL of people with depression. Third, our findings benefit our understanding of the psychopathological process of MDD symptoms affecting physical HRQoL. Our findings illustrated that for Chinese people with significant somatic symptoms, their psychological problems need also to be recognized.

There are several study limitations that need to be recognized. First, our findings might be limited because we used an earthquake-exposed sample. Thus, samples exposed to other trauma types should be used to further replicate our findings. Second, the limitation of self-reported symptoms has been acknowledged [60]. Therefore, additional replications with samples measured by clinical-administered MDD instruments are warranted. Third, variables which was used to evaluate the MDD model’s external validity in this study was limited. The external validity of this MDD model should be evaluated with other types of variables which related to MDD in the future studies. Fourth, the cross-sectional design has certain limitations in explaining causality [61]. This needs further verification by future longitudinal studies.

## Conclusion

Survivors of natural disasters typically have a high MDD prevalence, and accurately assessing MDD symptoms among this population is very important for post-disaster mental health care. The present study found that MDD could be best represented by the two-factor model in Chinese natural disaster survivors, and the MDD factors have different relationships with HRQoL. Our findings could help to better understand the different components of MDD among natural disaster victims, which could guide more sophisticated assessment and intervention for their MDD symptoms. Moreover, findings of the current study are important in contributing to improving HRQoL of natural disaster victims.

## Data Availability

The datasets used and/or analyzed in the current study are available from the corresponding author upon reasonable request.

## Abbreviations

MDD: Major depressive disorder
PHQ-9: Patient health questionnaire-9
SF-36: Short-form health survey
HRQoL: Health-related quality of life
CFA: Confirmatory factor analysis
SEM: Structural equation modelling
CFI: Comparative fit index
TLI: Tucker-Lewis index
RMSEA: Root-mean square error of approximation
